# Exploring Rural Children’s Ideal Healthy Smoothie: A Visual Focus Group Study

**DOI:** 10.3390/foods15203653

**Published:** 2026-10-15

**Authors:** Tennessee Randall, Danni Walters, Natalie Rouse, Tomos Owens, Amanda J. Lloyd, Louise Neilson, Laura L. Wilkinson

**Affiliations:** 1 School of Psychology, Faculty of Medicine, Health and Life Science, Swansea University, Swansea SA2 8PP, UK; 2 Twisted Orange, 14 Ballard Grove Sidford, Sidmouth EX10 9EP, UK; danni.walters@uea.ac.uk (D.W.); 2356500@swansea.ac.uk (N.R.); louise@twisted-orange.co.uk (L.N.); 3 School of Psychology, Faculty of Social Sciences, University of East Anglia, Norwich NR4 7TJ, UK; 4 Smwddis Swig Smoothies CYF, Elm Croft, Campbell Road, Caernarfon LL55 1BG, UK; tom@swigsmoothies.co.uk; 5 Department of Life Sciences, Aberystwyth University, Aberystwyth SY23 3DA, UK; abl@aber.ac.uk

**Keywords:** children, primary school, anticipated acceptance, smoothie, fruit, vegetables, co-creation, sensory properties, drawing tasks, theory of planned behaviour

## Abstract

Children can increase fruit and vegetable intake by consuming smoothies. However, most commercial smoothies contain excessive sugar due to the high proportion of apple juice within recipes. Including children, particularly those from potentially under-served communities, within the smoothie innovation journey is essential to develop healthier recipes that are also acceptable. Therefore, this study examined children’s perceptions of healthier smoothie base ingredients using a visual focus group methodology (i.e., creative drawing tasks with group discussions) that we have recently adapted for the food and drink industry context. Children attending a rural primary school (*n* = 28; 60.7% male) completed drawings on their current smoothie drinking behaviours, their ideal healthy smoothie, and barriers to consumption. Smoothies typically contained strawberries, bananas, blueberries, dairy-based milk, and ice, as well as calorically dense ingredients (e.g., ice cream and chocolate). Children’s ideal healthy smoothie contained fruit, ice and dairy-based milk, and the inclusion of vegetables, too many ingredients, a lumpy texture, and a large portion size were reported barriers. These findings highlight dairy-based milk, ice and whole fruit as potential ingredients for future product formulations, as well as the importance of co-creation and the effectiveness of visual focus groups as a method to elicit children’s ideas.

## 1. Introduction

Dietary patterns of children living in the UK are nutritionally insufficient in many areas. Firstly, only 19% of 5–15-year-olds meet the recommended minimum intake of five portions of fruit and vegetables per day [1,2]. Indeed, children consume 3.3 portions per day on average [2]. Another nutrient that is commonly under-consumed is fibre. Fibre has various functions within the body, and 2–18-year-olds should aim to eat 15–30 g/day [3,4,5]. Yet only 14% of 4–10-year-olds and 4% of 11–18-year-olds get enough fibre from their diet [6]. Additionally, children consume an excessive amount of ‘free sugar’. Free sugars refer to sugars added to food or those naturally present in juiced or pureed fruits and vegetables [7]. By contrast, sugars from whole fruit, vegetables and dairy products are not considered free sugars [7]. Free sugars should not exceed 5% of total energy intake [6]. However, most children and adolescents are estimated to consume more than double the recommended amount [6]. These findings highlight that children living in the UK generally consume a diet that is nutritionally insufficient and the areas which require considerable attention are fruit and vegetable intake, fibre intake, and free sugar consumption.

Dietary patterns have notable implications for health and wellbeing during childhood and adulthood. For example, previous research identified that children’s fruit and vegetable intake was negatively associated with Crohn’s disease risk in childhood and cardiovascular disease risk in adulthood (i.e., as a result of intake during childhood) [8,9], asthma and allergy symptoms in children and adults (e.g., wheezing) [10,11,12], antisocial and behavioural difficulties in children [13], pro-inflammatory biomarkers in children and adolescents [14], immune function in children [15], depression symptoms in children and adolescents [16] and higher energy intake later in childhood [17].

By contrast, fruit and vegetable intake was not related to children’s weight or body mass index [18,19,20,21,22,23]. Free sugar intake, particularly from beverages, was positively related to the risk of overweight and obesity in children [24,25,26,27,28,29,30]. Other identified issues relating to increased sugar intake in children included poorer dietary quality [31,32,33,34], and an increased risk of dental caries (i.e., tooth decay) [35,36,37]. Regarding fibre, a previous review reported that higher intake in children was associated with better cholesterol levels during childhood, lower body mass index in adulthood and improved responses to insulin in adolescence [38]. Generally, the evidence demonstrates that children’s dietary patterns influence many short-term and longer-term health outcomes and strategies should thus focus on increasing consumption of fruit, vegetables and fibre whilst reducing free sugars.

One strategy aimed at addressing fruit and vegetable intake is smoothies. Smoothies are blended beverages that can contain fresh and/or frozen fruits, vegetables as well as other ingredients [39]. Previous studies have demonstrated that smoothies can increase fruit and vegetable intake and are highly liked by children. For example, one study reported that the number of fruit servings consumed by children increased by 54%, over a 10-week period, following the introduction of fruit smoothies during school breakfast programmes [40]. Another recent study evaluated a 4-week nutrition programme at a rural school [41]. The children consumed four different smoothies during the programme and were educated on the functional role of the smoothie ingredients within the body. Ratings indicated that 72.8% of children either “loved” the smoothies or rated them as “good” on average, and the children reported a high willingness to consume the smoothies again. Alternatively, the study in [42] focused on the inclusion of dark green vegetables (e.g., spinach, kale, collards) within smoothies that were served to 3–5-year-olds. Notably, 84.3% of the children willingly tried the various green smoothies and ad libitum intake did not significantly differ between children who drank a smoothie with fruit and vegetables and children who drank a smoothie with fruit only. These findings indicate that smoothies can address key barriers to the consumption of whole fruit and vegetables in children, such as taste (e.g., bitter, sour, bland), texture (e.g., mushy, slimy, gooey) and appearance (e.g., unripe, weird, wrinkled) [43].

Although smoothies have the potential to increase fruit and vegetable intake, concerns have been raised over free sugars within commercially available smoothies. For instance, one report compared commercial fruit juices, juice drinks and smoothies marketed for children and reported that smoothies contained the highest amount of sugar on average [44]. Furthermore, 95.8% and 70.8% of smoothies exceeded the maximum recommended daily amount of free sugars for 4–6-year-olds (i.e., 19 g) and 7–10-year-olds (i.e., 24 g), respectively [45]. Similarly, another study found that smoothies contained more calories than commercial fruit juices and juice drinks and there was a strong correlation between sugar and energy content, indicating that beverages with increased sugar also had more calories [46]. Another concern relates to portion size. According to a previous report [47], most smoothies sold in supermarkets contained 250–400 mL, which exceeds the recommended portion size of 150 mL [48]. The high sugar content of commercial smoothies has been suggested to be driven by the predominant use of apple juice within industry recipes. Indeed, an analysis of eight commercial smoothies indicated that the proportion of apple juice used in recipes was 57.4% on average [49]. Apple juice is a highly affordable ingredient that is easily processed, stored and transported [50,51]. Although these attributes are highly desirable for the food industry, they have resulted in the ‘applejuicification’ of commercial smoothies, which are characterised as being sugary and highly palatable [52]. Consequently, the sugar content of commercial smoothies, especially those marketed for children, must be addressed to reduce children’s intake of free sugars.

Reformulation is one strategy that the food industry can utilise. In brief, reformulation is the process of redesigning an existing product with the aim of developing a healthier product [53]. Some approaches to reformulation include (1) reducing the amount of a specific nutrient, such as free sugars or saturated fat, (2) increasing the amount of a nutrient, such as dietary fibre, (3) including vitamins and minerals to support intake of micronutrients and (4) reducing the portion size of products [54]. The literature has supported the potential of reformulated food and beverages to reduce sugar intake and improve health outcomes [55,56,57,58]. Although, few studies have examined reformulation in the context of fruit smoothies. One study reported a lower fat and calorie content in smoothies made with puréed bananas and orange-fleshed sweet potato, compared to a control smoothie made with plain yoghurt and sugar [59]. Rated sweetness and acceptability were also similar between smoothies, which highlights the potential for healthier ingredients in smoothies. However, the participants in this study were adults. Consequently, the findings may be less generalisable to children as sensory preferences and acceptability have been shown to differ between children and adults [60,61,62,63,64]. Besides improved nutrition, cost could be another motive for the industry to reformulate smoothies. For instance, a Mintel report found that rising fruit prices negatively impacted the volume of fruit juice, juice drinks and smoothies sold in the UK and most surveyed consumers indicated a preference for brands to change their recipes in response to rising fruit prices [65]. Taken together, product reformulation has the potential to improve nutrition and brand loyalty in response to rising ingredient costs.

Yet a key challenge to the success of healthier product reformulation is consumer acceptance. If reformulated products are perceived to be substantially different from the original formulation, then the new product is at risk of being rejected [53]. Indeed, the reformulated product should be rated the same (as a minimum) as the original, especially for taste [66]. This was evident in a previous pilot study that examined the effect of a berry-enriched smoothie on children’s attention span and concentration in school [67]. Using a cross-over trial, the children consumed two smoothies: the berry-enriched smoothie (ingredients: pear, apple, strawberry, blackcurrant, carrot, elderberry, spinach, oat), that contained high levels of vitamins and minerals, and the control smoothie (ingredients: water, sugar and fruit juices). Prototypes of the berry-enriched smoothie were initially evaluated by an ‘internal expert panel,’ followed by preference testing in school children with three prototypes. Acceptance tests, hedonic scales, and ranked preferences informed which prototype was tested in the pilot trial. However, compared to the control smoothie, the children consumed significantly less of the berry-enriched smoothie during the trial and their rated liking was lower.

One possibility is that a smoothie that is co-created with children may allow for a reformulation with health in mind that mitigates this risk of consumer rejection. Indeed, previous research with children and adolescents has successfully utilised co-creation to inform the development of healthy and sustainable food products and beverages [68,69,70,71]. Therefore, our approach was to co-create ideas with children to design a healthier smoothie that also incorporated their taste and flavour preferences, using a protocol we recently developed based around the ‘visual focus group’ optimised specifically for the food and beverage context [72]. The visual focus group is a qualitative research method, featuring an art-based drawing task that is completed within the structure of a traditional focus group [73]. Drawing tasks can elicit creativity, which may encourage participants to think differently and thus avoid clichéd or ‘ready-made’ answers [74]. Furthermore, the method is inclusive for people who experience language or verbal communication barriers [72,73]. Drawings have been shown to facilitate communication in children [75,76]. Yet, few studies have applied this to food preferences [77,78]. The cited studies reported that the children’s drawings provided an additional layer of understanding, such as their ideal variety (e.g., colour) and appearance (e.g., a perfectly shaped carrot) for fruit and vegetables. Additionally, the drawings were particularly helpful when children could not verbally identify or remember the name of target foods.

Consequently, the primary objective of this study was to apply our visual focus group methodology to establish children’s perceptions and anticipated acceptance of smoothies made with base ingredients that offer a viable alternative to the apple juice base that is predominantly used within the food industry, to promote a positive change in children’s health by increasing intake of fruit, vegetables and fibre and inform future formulations designed to reduce added/free sugars. Secondly, it is notable that access to healthy food is poorer in rural areas compared to urban areas [79,80,81]. Therefore, our secondary objective was to focus on the perspectives of children living in rural areas.

## 2. Materials and Methods

### 2.1. Participants

Our industry partner (Swig Smoothies) identified eight candidate primary schools for the visual focus group. The Headteacher of each school was contacted via email and was informed that the research team at Swansea University were recruiting children in years five and six to participate in an art-based workshop on healthy smoothies. The email also contained a ‘gatekeeper’ information sheet and ‘gatekeeper’ consent form. One primary school in a rural area of North Wales responded and provided consent for us to conduct the visual focus group. Subsequently, parental information sheets and consent forms were distributed to the parents of 28 children across two classrooms, informing parents that the study purpose was to use art to understand children’s preferences for smoothies and what a healthy smoothie looked like to them. The information sheet and consent form were also translated into Welsh for parents whose primary language was Welsh. As the research topic was neutral and the children did not consume any food during the visual focus group, there were no exclusion criteria, meaning that all children were eligible to participate. All parents provided consent for their child to participate, and the final sample consisted of 28 children. Their ages ranged from nine to 11 years old (M = 9.93; SD = 0.54) and most were boys (*n* = 17). Formal assent was not obtained from the children, but we ensured that another classroom was available for children who may not have wanted to participate in the visual focus group, though this was not required as all children participated in the entire session.

Information power was used to support the sample size for the visual focus group [82]. This approach states that the number of participants required for qualitative research depends on the extent to which the information held by the sample is relevant to the study. This suggests that fewer (more) participants are required if they hold more (less) information power. Information power is determined by the: (1) study aim, (2) sample specificity, (3) use of established theory, (4) quality of dialogue and (5) analysis strategy [82]. Our assessment indicated that the study aim was narrow due to the specific focus on smoothies. Furthermore, we expected the children to have adequate knowledge about smoothies and previous experience with consuming smoothies. Also, an established theory was utilised to develop the task protocols and discussion guide (see Section 2.2). We also expected that the quality of dialogue would be increased by the researcher’s experience in conducting qualitative studies and the uncontroversial nature of the topic, which may have facilitated open and honest responses from the children. Based on these criteria, information power could be achieved with relatively few participants. Therefore, a sample size of 28 participants was considered sufficient to achieve adequate information power.

### 2.2. Data Collection Instruments and Procedures

The visual focus group aimed to use individual and collective drawing tasks, as informed by [73], to explore current behaviours relating to smoothie consumption as well as children’s perceptions of their ideal healthy smoothie. The theory of planned behaviour (TPB; previously identified as a suitable framework for exploring influences on healthy eating behaviours [83,84,85]) informed the protocol for the drawing tasks and subsequent focus group discussions. This project was collaborative in nature. All partners, including industry partners, had an opportunity to contribute to the study via regular group meetings. We note that the industry partners did not place any limitations or restrictions on the research design. The protocol and discussion prompts were not pilot tested prior to the visual focus groups but were based on examples from previous visual focus groups.

The visual focus group lasted one hour. Ethical approval for this study was granted by Swansea University’s School of Psychology Study Ethics Committee (Approval number: 2 2024 11385 10692) in December 2024. The children were provided with suitable art materials for all drawing tasks, which included paper (A4, A3), pencils, and crayons. Turning to the personal characteristics of the research team, two researchers conducted the visual focus group (TR, PhD candidate & NR, PhD candidate). Both researchers were female. TR was employed as a research fellow, and NR was employed as a research assistant during the time of the study. Both researchers had previous experience with conducting visual focus groups. A relationship was not established with the children prior to the study commencement, but the teacher was present during the visual focus group, which facilitated rapport with the children. We addressed potential biases by recognising how our personal interests in the factors that influence the consumption of a healthy and environmentally sustainable diet could have influenced our responses to the children during discussions. The visual focus group was conducted in January 2025. Firstly, the children were introduced to the topic of smoothies and the approach of using drawings to communicate their ideas. The introduction was kept brief to limit potential demand characteristics. The session began with an individual drawing (Task one) of the smoothies that children currently consumed (i.e., behavioural component of the TPB). The children were prompted to draw the individual ingredients as well as the final ‘blended’ smoothie. After task one, the children were asked to explain their drawings to the researchers, and their responses were noted. The second drawing was completed in small groups (seven groups with four children per group) to explore subjective norms and behavioural intentions towards consuming a ‘healthy’ smoothie (Task two). The number of children per group was informed by recommendations that small groups are needed for art-based focus groups [73]. Each group was given a sheet of paper which depicted an outline of a blender. The children were instructed to collectively draw what they would include if they were to make a healthy smoothie and what that would look like to them. The children were also instructed that the ingredients they chose should also be enjoyed by the other children in their group. For this task, we did not define what a healthy smoothie was in order to elicit the children’s personal meanings and perceptions within the drawings. The third drawing was devised to explore perceived behavioural control (PBC). This task considered the barriers or challenges children experienced when drinking healthy smoothies (Task three). Following task three, the children participated in a group discussion to discuss the drawings of their ideal healthy smoothie and the barriers to drinking a healthy smoothie. One of the researchers moderated the discussion (TR) and the other researcher took notes throughout the drawing tasks and discussions (NR). This discussion was audio-recorded and transcribed to support the interpretation of the children’s drawings. At the end of the visual focus group, the children were thanked and given a notebook and pen for their contribution. Also, a debrief form was provided for parents which detailed that the study aimed to identify healthy smoothie recipes that were also enjoyable for children. The COREQ (COnsolidated criteria for REporting Qualitative research) Checklist is included in the Supplementary Materials. This was utilised to support thorough and comprehensive reporting on our qualitative methodology.

### 2.3. Data Analysis

The drawings were analysed using a combined critical visual discourse analysis [86] approach using content analysis (descriptive), semiotics, and iconography (connotative) [87,88]—adapted from [72] which also included a thematic analysis of the transcript of the group discussion. Firstly, for the content analysis, a researcher (DW, PhD candidate) reviewed the drawings for each task and counted the number of times a particular visual element appeared (e.g., total number of times a strawberry was drawn in task one). This step provided a quantitative background map as to what types of elements were more or less prominent in the drawings. A total of 28, 7 and 19 drawings were analysed for tasks one, two and three, respectively. In task one, the drawings were completed individually, whereas the drawings for Task two were completed collectively. In task three, the children were given the option to complete the task individually or collectively. None of the drawings were excluded from the data analysis and no specialist software was used to analyse the data. Secondly, a semiotic and iconographic analysis was conducted which built on the content analysis and provided qualitative detail in interpreting the meaning behind the elements (e.g., the prominence of ice cubes in the drawings suggested that the children thought it was important for a smoothie to be cold). To aid interpretation during this stage, the researcher (DW) also referred to the notes taken during the drawing tasks to support a deeper interpretation of the children’s reasons that underpinned their drawings (e.g., supporting the interpretation that it was important for the smoothies to be ice cold).

Combined, these approaches provided both a breadth and depth of analysis and enabled rich insights into the data. The results were collated into themes, following the structure of the three tasks, guided largely by the content analysis with the semiotic and iconographic analysis providing interpretive detail.

Two members of the research team (DW and TR) engaged in regular discussion during the analytic process in order to build consensus and ensure the adequate exploration of different and rich interpretations of the data. The children were not given a participant number. Therefore, the quotations presented in the results are anonymous.

## 3. Results

### 3.1. Composition of Typical Smoothies

**Figure 1:**
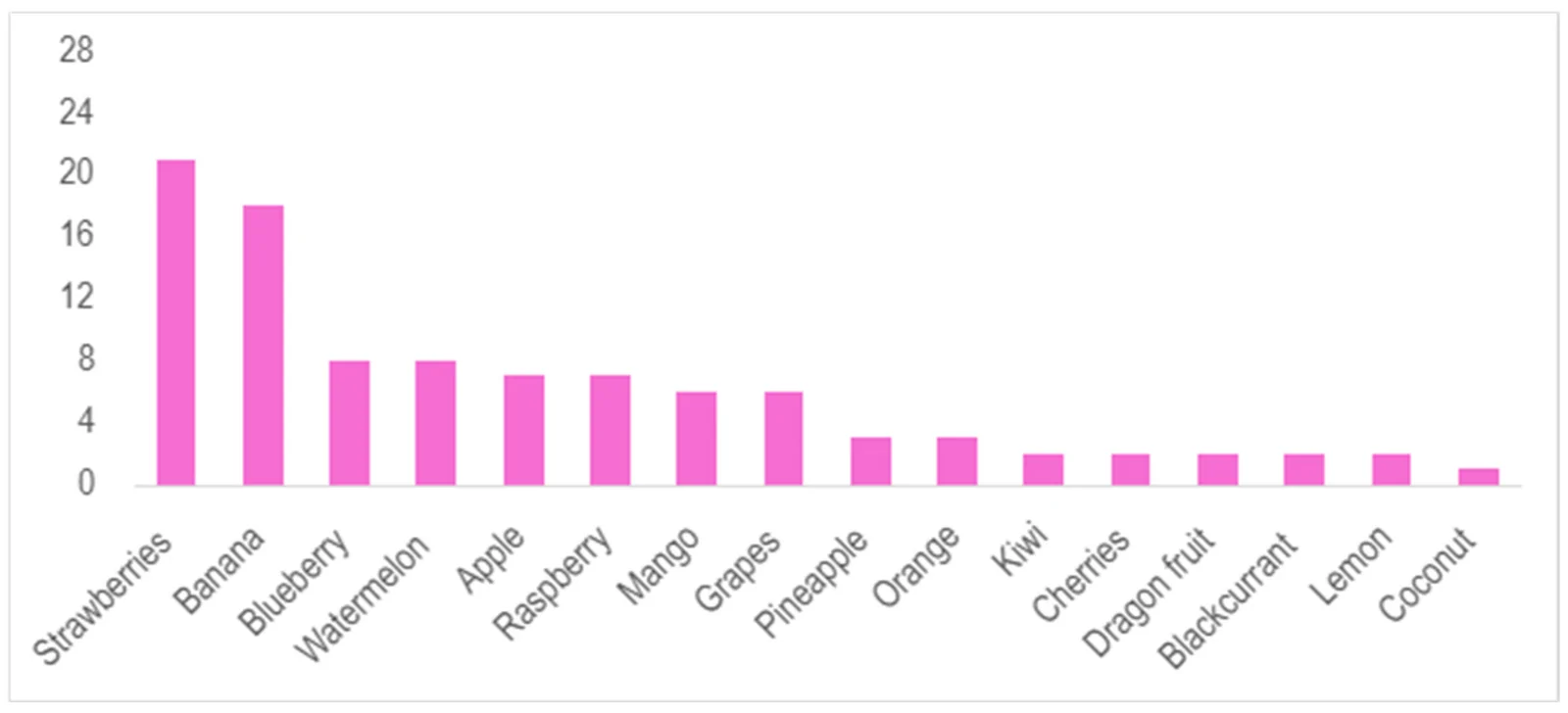
A summary of the fruit children typically included in smoothies.

Task one considered the smoothies that children typically consumed. Fruit was an important ingredient within smoothies. Across 28 individual drawings, the children drew 16 different types of fruit. The frequency with which each fruit appeared across drawings is summarised in Figure 1. Strawberries were the most frequently depicted fruit (*n* = 21), followed by bananas (*n* = 18), blueberries (*n* = 8), and watermelon (*n* = 8). Fewer drawings included exotic fruits (e.g., coconut, dragon fruit, kiwi). Generally, the children reported that familiarity was a key factor that influenced their fruit choices within smoothies.

**Figure 2:**
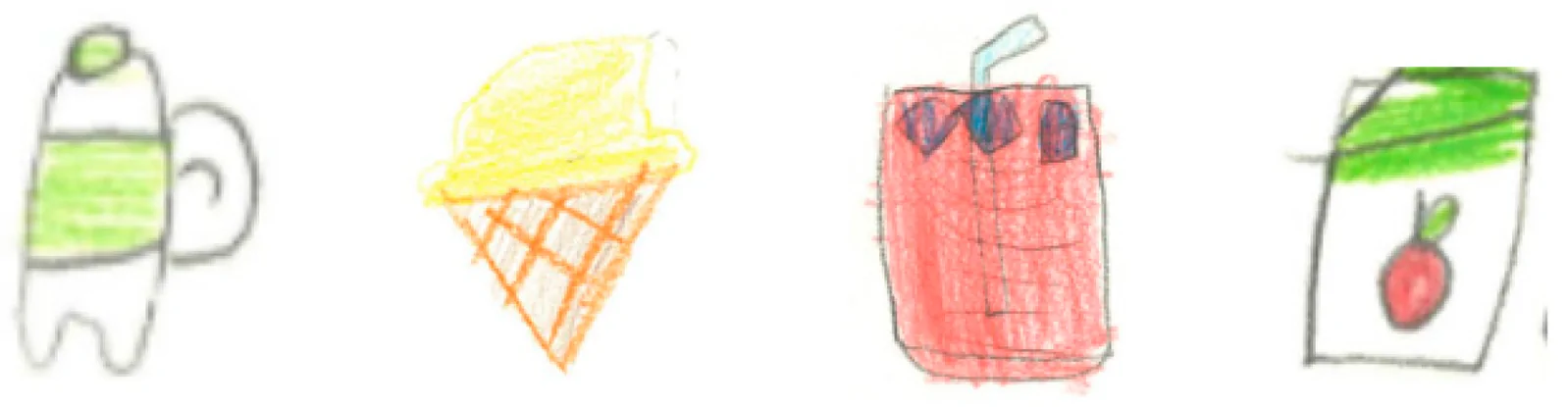
Example drawings of the different base ingredients, from left to right: milk; ice cream; ice and apple juice.

Across 28 individual drawings, the children drew six different types of base ingredients, which included dairy-based milk (*n* = 7), ice cream (*n* = 5), ice (*n* = 4), fruit juice (*n* = 2), and yoghurt (*n* = 1). Ice was often drawn as floating in the glass, emphasising a desire for the smoothie to be cold. Some of the drawings included multiple base ingredients, such as ice cream and milk (*n* = 3), ice and milk (*n* = 1), ice cream, milk and ice (*n* = 1), ice and ice cream (*n* = 1) and yoghurt and ice (*n* = 1). See Figure 2 for a representation of base ingredients across drawings.

Additionally, some drawings included extra ingredients that were perceived to increase the sweetness and creaminess of smoothies that were typically consumed. Chocolate (*n* = 6) and cream (*n* = 6) were the most frequently depicted added ingredients. Some of the children also referenced well-known chocolate brands. Cream was typically depicted as having a whipped appearance and was placed on top of the smoothie. A few drawings (*n* = 3) also included sprinkles on top of the cream. One child drew confectionery sweets and another included honey as a sweetener.

The drawings shared characteristics relating to the appearance of a typical smoothie. For example, most drawings (*n* = 18) depicted the smoothie in a glass or takeaway cup with a straw. Straws were considered an integral component of the smoothie. Indeed, one child stated that:

*“If I saw a smoothie without a straw, I’d probably see it as a juice, the straw just gives off a smoothie vibe”*.

**Figure 3:**
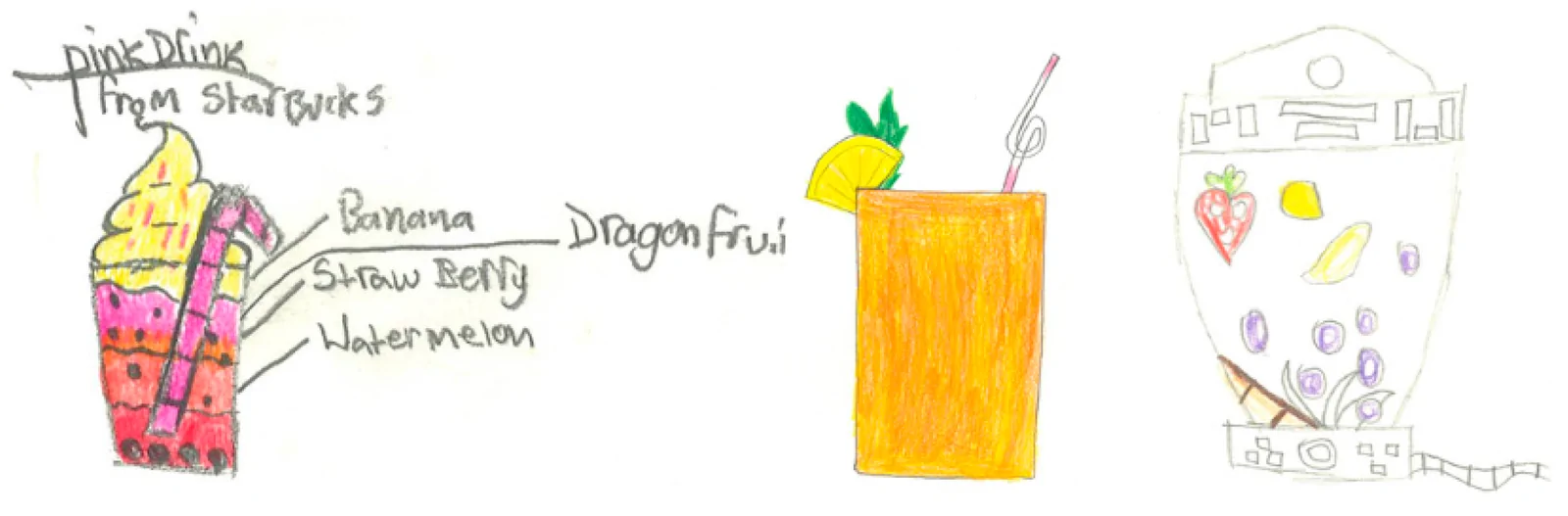
Example drawings of the appearance of typically consumed smoothies, from left to right: smoothie obtained from a venue outside of home; a smoothie in a glass with a straw; a blender containing bespoke smoothie ingredients.

Additionally, the depiction of smoothies within takeaway cups reflected how smoothies were at times consumed outside of the home. Indeed, some of the children specifically mentioned mainstream coffee shops and fast-food chains. On the other hand, some drawings (*n* = 4) depicted blenders that contained the individual smoothie ingredients, highlighting that these children typically associated smoothies with fresh and bespoke ingredients. See Figure 3 for a representation of the typical appearance of smoothies across drawings.

### 3.2. The Ideal Healthy Smoothie

Task two considered children’s perceptions of their ideal healthy smoothie. As a group, the children discussed their ideas and then created drawings that represented their collective ideas as a group. Similar to Task one, fruit was a key component within healthy smoothies. Across the seven drawings, strawberries (*n* = 6), bananas (*n* = 5), blueberries (*n* = 3), raspberries (*n* = 2), and watermelon (n = 2) were the most frequently included fruits within drawings. Most drawings (*n* = 4) depicted multiple fruits within healthy smoothies.

**Figure 4:**
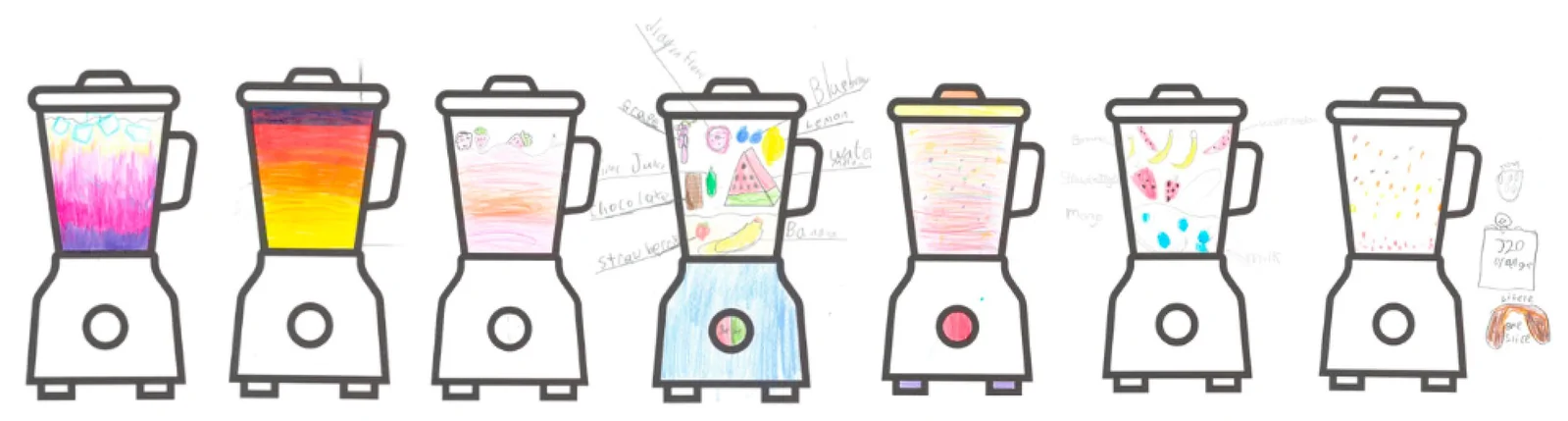
Children’s collective drawings of their ideal healthy smoothie.

Ice (*n* = 4), milk (*n* = 2), orange fruit juice (*n* = 2), coconut milk (*n* = 1), and vanilla ice cream (*n* = 1) were depicted as base ingredients. Considering extra ingredients, chocolate was included in some drawings (*n* = 2). One of the groups emphasised in their drawing that a small portion of chocolate was appropriate for their ideal healthy smoothie, using words such as “little” and “one slice [of chocolate]”. Also, one drawing depicted a scoop of protein powder. Alternatively, some drawings emphasised the visual appearance of the smoothie, as opposed to the individual ingredients, which was often depicted with bright colours, as well as ombre effects. See Figure 4 for a depiction of the ideal healthy smoothies across the groups.

Discussions revealed various sensory-related factors that were important for children’s anticipated acceptance of healthy smoothies. For instance, the children stated that smoothies should contain a variety of ingredients. This was underpinned by perceptions that increasing the number of ingredients would improve the smoothie flavour and reduce bitterness:

*“If there was only one fruit it would taste more bitter”.*

Additionally, some children were specifically interested in trying novel flavours and one child reported a sense of uncertainty about whether combining ingredients improved flavour:

*“If you have one flavour, you’re already gonna know what it tastes like”.*

*“It’s gotta be like mixed up and maybe it will be nice, or maybe it will be not nice and you can have a try of them.”*

Several children associated smoothies with cooling and refreshing sensations. This was attributed to the inclusion of ice, which was perceived as an ingredient that maintained the cold temperature of the beverage and provided textural stimulation:

*“It looks like a sunset and it’s really refreshing and sometimes I just eat the ice… its refreshing, cold and I love to bite it”.*

Texture was a crucial factor for acceptance. Specifically, smooth textures were strongly preferred, and this was perceived to facilitate children’s consumption:

TR: *“So why is it important for it to be smooth”.*

*“So you don’t choke, you don’t get lumps, it’s easier to swallow and the texture is better”.*

### 3.3. Barriers or Challenges to Consuming Healthy Smoothies

Task three considered children’s perceived barriers to drinking healthy smoothies. Similar to Task two, sensory-related factors were prevalent within discussions. For example, one child indicated that the liquid colour was important for their overall evaluation of the smoothie:

*“If the smoothie was green it would look weird… it would taste weird”.*

Another child stated that the inclusion of vegetables reduced the visual appeal of smoothies:

*“I put vegetables in mine, like a lot of vegetables and it kinda looks like baby food”.*

In contrast, another child stated that the smoothie appearance was less important than the taste:

*“I think, probably I’m saying this because I like the ones that I make but I don’t really care about how they look, I just care about how they taste”.*

Vegetables were generally considered a major barrier for the taste of healthy smoothies. Broccoli, peas, avocado and cucumber were depicted as strongly disliked ingredients and would often be accompanied by negative icons, such as a sad face or a red circle with a diagonal line (i.e., no symbol). According to the children, vegetables were “*too bland*” for smoothies. One child mentioned that vegetables were less appropriate for smoothies because they reminded them of a dinner meal:

*“If I had vegetables in a smoothie, it’d give me off like a dinner vibe… it would make you feel like you have no teeth and then you blend up your food and then you eat it like a soup dinner”.*

Although, one child was more receptive to the inclusion of spinach because it “*doesn’t really have any taste, so it wouldn’t be actually that bad*”.

Other ingredients that reduced children’s anticipated acceptance of healthy smoothies were lemon juice, beans, honey, ginger, cherries, and raisins.

Despite the desire for sweetness and variety in the previous tasks, excessive sweetness and flavours were also a reported barrier to healthy smoothies:

*“There could be too many things that could make you sick… like too many fruits and the flavour goes”.*

As mentioned previously, smoothie texture was crucial for anticipated acceptance and the children were concerned that including seeds, nuts or ingredients with ‘bits’ would result in a chunky or lumpy texture due to the smoothie not blending properly. This was problematic for the children because it meant that the smoothie was more difficult to drink with a straw:

*“I wouldn’t like the seeds getting stuck in my teeth”.*

*“It depends on what kinds of seeds… if they are like big or small”.*

**Figure 5:**
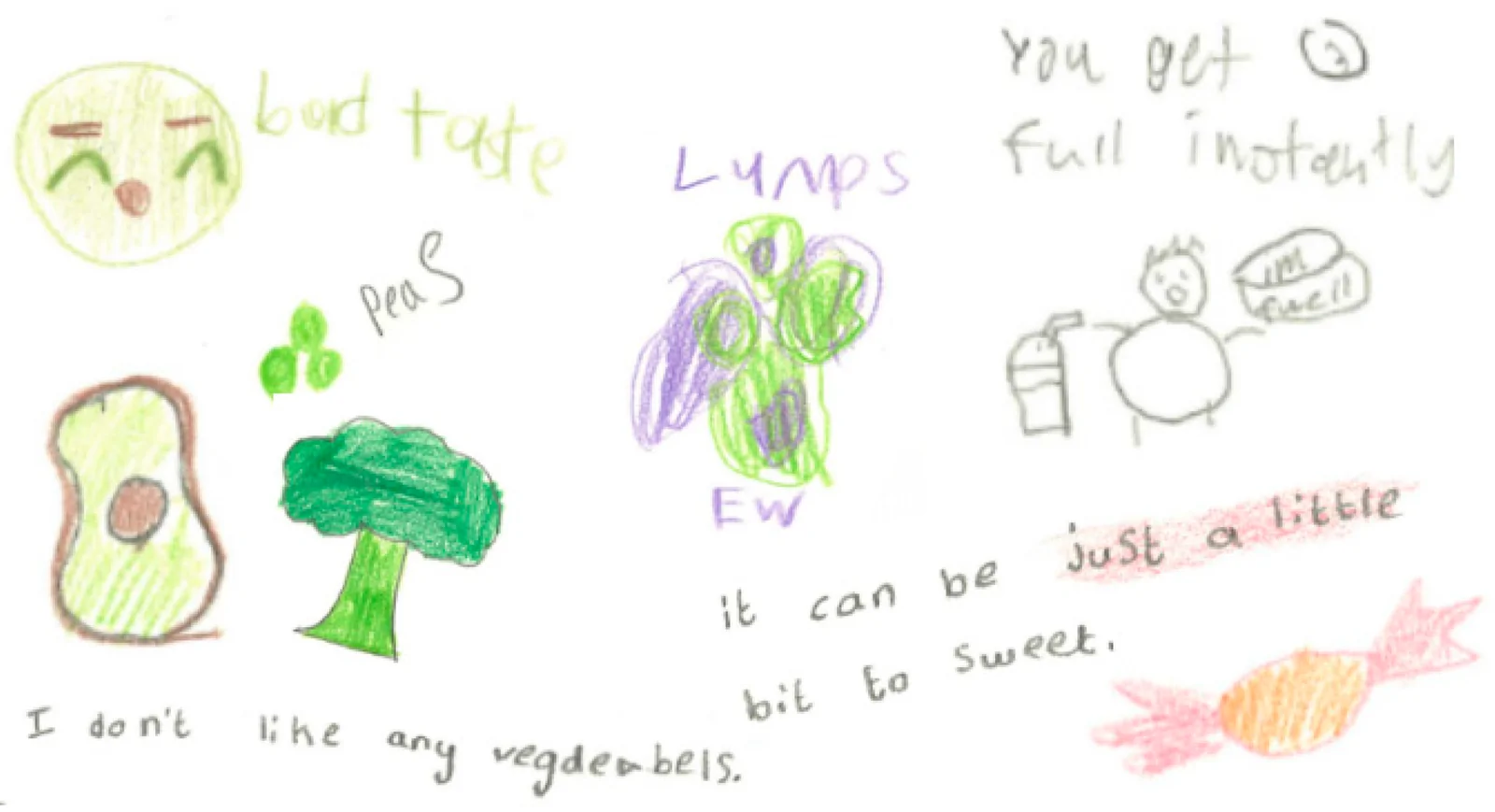
Children’s drawings of the barriers to drinking healthy smoothies.

According to stated smoothie preferences, portion size was also a critical factor. For instance, the children mentioned concerns with smoothies being too large, which could result in uncomfortable fullness or even sickness. Finally, the temperature of drinks was important as smoothies that were either warm or too cold were considered unenjoyable. See Figure 5 for a summary of the depicted barriers to healthy smoothies:

## 4. Discussion

Most children in the UK do not eat enough fruit, vegetables or fibre and consume excessive free sugars, which increases their risk of developing various health problems. Consequently, dietary strategies are essential to address nutritional deficiencies. One strategy to increase children’s consumption of fruit and vegetables is through the provision of smoothies. However, most commercially available smoothies have excessive sugar due to the high percentage of apple juice used in recipes. The food industry could address this through reformulation but there is a risk that reformulated products will be rejected if the taste substantially differs from that of the original. Nevertheless, co-creating smoothies with children could mitigate this risk. Therefore, this study aimed to establish anticipated acceptance and perceptions of healthier smoothie bases with children living in rural areas, who experience additional barriers to accessing healthy food, compared to children living in urban areas. We applied our visual focus group methodology to explore current smoothie drinking behaviours, the ideal healthy smoothie, and barriers to healthy smoothies. Smoothies that were typically consumed contained strawberries, bananas, blueberries, dairy-based milk, and ice, as well as calorically dense ingredients (e.g., ice cream, whipped cream, and chocolate). Key ingredients in the ideal healthy smoothie were whole fruit, ice, and dairy-based milk. Barriers to healthy smoothies were the inclusion of vegetables, too many ingredients, a lumpy texture, and a large portion size.

Our findings support the importance of familiarity as a driver of children’s food preferences [89]. Indeed, strawberries and bananas consistently featured in children’s drawings of their current and ideal healthy smoothie. A previous study also reported a high preference for these fruits in 3–5-year-olds from Finland [90]. The preference for familiarity can prevent children from experiencing negative consequences (e.g., nausea, vomiting) from eating potentially harmful foods [91]. Likewise, children have an innate preference for sweet tastes which persists during childhood, as well as an aversion to bitter tastes [92], which could explain differences in the positive and negative representation of fruits and vegetables within smoothies, respectively. Although, the children’s aversion to vegetables within smoothies contradicts previous findings that 84.3% of 3–5-year-olds willingly tried smoothies with various dark green vegetables [42]. Indeed, children in the current study believed that green vegetables would impact the colour, texture, and taste of smoothies and typically drew emotions relating to disgust, sadness, and nausea. Our interpretation of this discrepancy considers differences in information provision across studies. For instance, researchers in the previous study did not tell the children that their smoothies contained dark green vegetables [42]. Indeed, the smoothie was described as a “green drink that has fruit in it… like strawberries, pineapples and peaches”. Additionally, the smoothies were served in a covered soufflé cup with a straw, indicating that the ‘green’ appearance was likely to be masked. Therefore, the children’s willingness may have changed if they were aware of the ingredients. These findings have implications for the inclusion and marketing of vegetables within commercial smoothies. For instance, an industry report by Mintel found that parents and grandparents prioritise health (e.g., a good source of vitamins, low sugar) and their child’s preferences when buying food and drink products for children [93]. Therefore, labelling and promotion of fruit and vegetable smoothies must carefully balance these cues in order to appeal to both children and parents.

Smoothie texture was another highly important attribute. Specifically, the children desired a smooth texture because it was easier to drink with a straw and they disliked the idea of food particles, from seeds or certain fruits, in their teeth. These findings support a previous conjoint experiment that examined children’s food preferences. In this study, 88.5% of 9–13-year-old Danish children preferred images of smoothies without visible pieces of fruit, compared to images of smoothies with visible banana and raspberry pieces in the glass [94]. Furthermore, this preference persisted when children were presented with real smoothies. Similarly, another study found that children consumed significantly more yoghurt when it had a smooth texture, compared to yoghurt that contained small fruit pieces [95].

It has been suggested that children may experience disgust in response to ‘bits’ and ‘pips’ because they are viewed as a sign of contamination [91,96]. Another explanation for the rejection of certain textures considers children’s sensory processing abilities. For instance, food provides a multi-sensory experience (e.g., visual taste, touch, auditory) and children who experience heightened sensitivity to sensory stimuli may experience greater challenges with the taste, texture, temperature or smell of foods [96]. The aversion to specific food textures has implications for eating behaviour and dietary quality. For instance, a cross-cultural study reported that preferences for foods with a soft texture were associated with higher food neophobia and lower vegetable consumption in children, especially in the UK and Spain [97]. Although, there is also evidence that food texture preference is related to age, indicating that older children are more likely to accept foods with particles [96]. Despite this, the children in our study were older and still expressed an aversion to smoothies with particles. The difference in findings could be explained by familiarity, whereby children in the current study were more likely to have experienced smoothie textures without any particles. This is also supported by [96], which found that children who had previously tasted foods with particles (e.g., orange juice with pulp, chunky peanut butter, strawberry jam with pieces, strawberry yoghurt with pieces, tomato soup with pieces) were significantly more likely to prefer foods with particles.

Other desirable attributes of healthy smoothies included sweet taste, varied flavours and a small portion size. Children’s preferences for sweet-tasting foods are well established in the literature [98,99,100,101,102] and are estimated to persist until mid-adolescence [92]. Children’s innate preference for sweet tastes is suggested to be influenced by a requirement for energy-dense foods to support development and growth [103]. This may explain why children in the current study also favoured energy-dense ingredients, such as ice cream, cream, and chocolate. Indeed, it is of interest that these ingredients, which may not typically be considered healthy, were mentioned by the children, and this highlights the importance of understanding perceptions of potentially ambiguous concepts (i.e., healthy) that can also be context dependent.

Turning to variety, previous work suggests that food variety in children is initially limited from two and a half years to eight years, but then gradually increases as children are exposed to more foods and become more willing to try new foods [104]. The preference for variety may also differ between food groups [104], which could explain why children in the current study drew smoothies with a variety of fruits, whereas smoothies with any vegetables were disliked. Despite children’s preference for variety, too many flavours were also considered a barrier. These findings are similar to a previous experiment that compared children’s attention span and concentration in response to consuming a ‘berry-enriched’ (10 ingredients) or control smoothie (six ingredients) [67]. Notably, intake was significantly lower in the berry-enriched condition and only 18% of children rated the berry-enriched smoothie as ‘very tasty’, compared to 63% of children in the control condition. Consequently, the complexity and variety of ingredients used in the berry-enriched smoothie may have reduced acceptance. Further sensory tests are needed to determine the optimal smoothie flavour intensity for children and whether this varies across age groups. For instance, do younger children prefer smoothies with fewer ingredients.

Children’s anticipated acceptance of healthy smoothies was also influenced by portion size, whereby drinking a large smoothie was related to feelings of nausea and uncomfortable fullness. These findings contradict previous research which found that children preferred images of large smoothies (145 g), compared to small smoothies (65 g) [94]. In this study, the smoothies were depicted in a transparent glass that was either full or half-full to reflect the large and small portion, respectively. Considering this, the preference for a large smoothie could be explained by visual cues relating to a product’s ‘completeness’. For example, completeness can be described as the extent to which the object (e.g., smoothie liquid) equals one unit (e.g., a glass or cup) and a product is incomplete if its shape does not appear to be a full product (e.g., a sandwich that is already cut in half) [105]. One study demonstrated that consumers preferred products with a complete shape, compared to an incomplete shape [106]. Consequently, the ‘large’ portion may have been selected due to the preference for a full glass as opposed to a half-full glass. Children’s aversion to a large portion size in this study could be explained by expected satiation, which is defined as the extent to which a food is expected to produce a feeling of fullness immediately after consumption [107]. Previous research has shown that expected satiation can change following repeated exposures to a food and that this judgement is learned via an association that forms between the sensory characteristics of a food and its post-ingestive consequences [108]. Supporting this, another study reported a relationship between expected satiation and texture, indicating that dairy products with a thicker texture were also expected to be more filling [109]. Therefore, the children may have believed that drinking a large smoothie was associated with negative post-ingestive consequences (i.e., nausea), which was informed by their perceptions of the smoothie texture. Future research is needed to determine the precise portion that can deliver an acceptable level of satiation and nutrients to children and to identify potential age differences, which is important from the perspective of nutrient sufficiency and food waste.

Whilst this was not a traditional sensory study, sensory-related factors, such as taste, flavour, visual appearance, temperature and texture were of clear importance to the children throughout discussions and drawings. Our findings demonstrated that children precisely identified sensory attributes that influenced their anticipated acceptance of healthy smoothies. Indeed, children’s aversion to vegetables was driven by expectations that such ingredients would reduce the visual appeal and liking of smoothies. Nevertheless, interventions that utilise taste exposure and ‘flavour–flavour’ learning have been shown to increase children’s acceptance of vegetables [110,111]. This approach could be effective within the context of smoothies, as demonstrated by [42], and requires further examination in older children. Another study examined the influence of temperature on rated sensory attributes and emotional responses to soup samples [112]. The intensities of 26 emotional and 15 sensory attributes significantly differed with variation in sample temperature. Similarly, anticipated acceptance of healthy smoothies was influenced by temperature. Children in the current study expressed a desire for beverages to be cool and refreshing, whereas extreme temperatures (i.e., too high/low) were a barrier to consumption. Further research is needed to determine the optimal temperature of smoothies for children and the extent to which temperature influences sensory perceptions.

The main strength of our study was our methodological approach. This was the first time that our adapted visual focus group protocol was conducted with children, and our results demonstrated its effectiveness in engaging children on topics relating to healthy eating. Furthermore, our confidence in the inclusivity of our approach was strengthened by feedback from the teacher who supervised the classroom during the study. Indeed, the teacher expected some children not to participate due to shyness but was pleasantly surprised as these children were highly vocal and engaged during the task.

As well as strengths, we must also acknowledge the limitations of this research. Firstly, our sample consisted of a single-school convenience sample, and we did not collect any sociodemographic data (e.g., food access, quality of the food environment) about the children. This limits the generalisability of the findings to children in other rural areas of the UK or other children living in North Wales. Also, we could not determine whether living in a rural area influenced the children’s perceptions of smoothies. Future research could address this by recruiting children from rural and urban areas and comparing their responses. We also note that co-creation with children does not guarantee that subsequent products will be accepted [113]. Indeed, the children in this study did not consume any smoothies, so it is possible that their ideal healthy smoothie may not meet their perceived expectations. Considering this, sensory testing is needed to evaluate the visual, taste and oral tactile attributes of the ingredients and recipes identified in this study. Furthermore, a nutritional analysis would facilitate the development of smoothie formulations that balance children’s sensory and stated preferences, as well as their nutritional requirements.

Additionally, the study revealed aspects of the visual focus group that require further optimisation for a classroom setting. For instance, conducting the drawing tasks within the classroom meant that the children’s drawings could have been influenced by their peers, thus influencing socially desirable responses. Considering this, future studies that use this approach within schools may benefit from changing seating plans, if permitted by the teacher. Secondly, the time for group discussions was limited due to the class size, which meant that not all children had an opportunity to explain their drawings. Consequently, the interpretation of the drawings was more challenging when the children did not include annotations on their drawings. This could be addressed by including additional tasks to clarify children’s perceptions, as demonstrated by [114].

## 5. Conclusions

Smoothies can increase children’s fruit and vegetable intake, but the nutritional value of commercial smoothies is often degraded by the high proportion of apple juice. Sugar reduction is possible through recipe reformulation, but there is a risk that consumers will reject products if taste differences are detected. Nevertheless, co-creation has the potential to mitigate this risk by integrating children throughout the smoothie innovation journey. Therefore, we recruited 9–11-year-old children from a rural area in North Wales and applied our visual focus group methodology to examine their anticipated acceptance of healthy smoothies. Ice, dairy-based milk and whole fruits were identified as potential base ingredients that could be implemented within future product formulations. By contrast, the inclusion of vegetables, an excessive number of ingredients, a lumpy texture and a large portion size were key barriers to consuming a healthy smoothie. Our results could inform the development of prototypes in which small quantities of vegetables or alternative base ingredients are incorporated while maintaining acceptable sensory properties and transparent product labelling. Further sensory testing with smoothie prototypes is needed to identify the flavour, variety and portion size that deliver optimal taste and nutrition for children and to provide evidence for age differences, which can inform whether recipes should be tailored for specific age groups.

## Data Availability

The data presented in this study are available on request from the corresponding author due to privacy.

## Supplementary Materials

The following supporting information can be downloaded at: https://www.mdpi.com/article/10.3390/foods15203653/s1, COREQ (COnsolidated criteria for REporting Qualitative research) Checklist. Reference [115] is cited in the supplementary materials.

## Abbreviations

The following abbreviations are used in this manuscript:

TPB	Theory of planned behaviour
PBC	Perceived behavioural control
